# Cellular Oxidative Stress

**DOI:** 10.3390/antiox10030399

**Published:** 2021-03-06

**Authors:** Silvia Dossena, Angela Marino

**Affiliations:** 1 Institute of Pharmacology and Toxicology, Paracelsus Medical University, Strubergasse 21, 5020 Salzburg, Austria; silvia.dossena@pmu.ac.at; 2 Department of Chemical, Biological, Pharmaceutical and Environmental Sciences, University of Messina, Viale F. Stagno D’Alcontres 31, 98166 Messina, Italy

Oxidative stress on a cellular level affects the function of tissues and organs and eventually of the whole body. The Special Issue “Cellular Oxidative Stress” has been conceived to collect and contribute to the dissemination of novel findings unraveling the impact of oxidative stress on cells, their subcellular components, and biological macromolecules. Great emphasis is placed on the effects of oxidative stress on erythrocytes and neurons and oxidative-stress-related conditions, including cardiovascular diseases, cancer, aging, and inflammation. In this context, the potential beneficial effects of natural and synthetic antioxidants have also been considered. Here, we offer an overview of the content of this Special Issue, which collects 17 original articles and nine reviews.

Both nucleated and anucleated cells have widely been used to study the effects of oxidative stress and antioxidants on a cellular level. Specifically, erythrocytes are continuously exposed to circulating oxidant molecules; oxidative stress associated to hyperglycemia represents an additional threat to cell homeostasis. Morabito et al. showed that the function and the expression of Band 3 protein (B3p), one of the most peculiar erythrocyte proteins, are affected in erythrocytes from diabetic subjects, and these effects were linked to the production of glycated hemoglobin (Hb) as well as oxidative stress. As oxidative stress, but not glycated Hb, was observed following the exposure of erythrocytes to increased glucose concentrations in vitro, these authors suggested that oxidative stress, rather than glycated Hb, is the key factor leading to early detrimental changes in poorly controlled hyperglycemia [1].

B3p has also been the focus of a study evaluating the effect of d-Galactose (d-Gal) on the anion exchanger activity. Despite d-Gal being known to induce oxidative stress, exposure of erythrocytes to relatively low (0.1–10 mM) d-Gal concentrations led to a reduced anion exchange capability independent from oxidative stress, but rather linked to glycated Hb production. This study sheds light on the early effects of excessive d-Gal on membrane transport systems and possible complications related to undiagnosed galactosemia [2].

Extracellular vesicles (EVs) are continuously produced in human blood from different cell types including erythrocytes, and their formation is triggered by various factors, including exposure to reactive oxygen species (ROS) and aging-associated oxidative damage. Sudnitsyna et al. showed that, following oxidative stress, erythrocytes produce EVs containing hemoglobin oxidized to hemichromes. Oxidative stress led to caspase-3 activation and B3p clustering in cells and EVs, events that are normally linked to eryptosis and removal of senescent erythrocytes from the blood circulation. Based on these findings, the authors suggested that erythrocytes might eliminate damaged hemoglobin by vesiculation as a protective mechanism to prolong their lifespan during oxidative stress [3].

In erythrocytes infected by *Plasmodium falciparum*, oxidative stress induces the production of hemichromes, which contain partially denatured hemoglobin with reactive iron. In turn, oxidative stress generated by hemichromes contributes to the activation of artemisin, a component of the standard treatment for *P. falciparum* infection. Tsamesidis et al. showed that Syk kinase inhibitors increase oxidative stress in parasitized erythrocytes by inhibiting the release of hemichromes and synergize with artemisin in producing a toxic effect against the parasite. These authors suggested that Syk kinase inhibitors might represent a useful strategy to increase the efficacy of artemisin-based combination therapies, especially in resistant strains [4].

Neurons are particularly vulnerable to oxidative damage; it is, therefore, not surprising that ROS play a fundamental role in the exacerbation and progression of neurodegeneration. Non-coding RNAs respond to oxidative stress with changes in their cellular abundance and, in turn, regulate gene expression networks involved in ROS homeostasis and buffering. Gámez-Valero and co-authors summarized the current knowledge on the role of non-coding RNAs in Alzheimer’s disease, Parkinson’s disease, Huntington’s disease, and amyotrophic lateral sclerosis and suggested that these biomolecules might represent novel therapeutic targets in neurodegenerative conditions [5].

Oxidative stress in the central nervous system plays a fundamental role not only in neurodegeneration, but also in epilepsy. In *status epilepticus*, oxidative stress and neuroinflammation may lead to mitochondrial dysfunction, structural damage, and cell death. Lin et al. summarized the current knowledge supporting the use of antioxidants in epilepsy from animal studies as well as clinical trials [6].

Traumatic brain injury (TBI) is a major cause of death among youth in industrialized societies. The review “Management of Traumatic Brain Injury: From Present to Future” collects the current knowledge on TBI pathophysiology and examines existing and potential new therapeutic strategies, including the use of antioxidants, in the management of inflammatory events and behavioral deficits associated with TBI [7].

Barancik and co-authors reviewed the molecular mechanisms of the antioxidant and anti-inflammatory activities of molecular hydrogen in the cardiovascular and central nervous system and dissected the corresponding effects on intracellular signaling pathways, gene expression, and autophagy. Molecular hydrogen exerts a dual action in protecting cells against oxidative stress by directly reacting with ROS and inducing the cellular antioxidant machinery. These authors suggested that the therapeutic potential of molecular hydrogen in pathological conditions including ischemia-reperfusion injury, brain edema, and neurodegeneration deserves to be further investigated [8].

Oxidative stress plays a crucial role in cardiac and vascular abnormalities in different types of cardiovascular diseases. Han et al. summarized the current knowledge on how oxidative stress could contribute to the pathogenesis of one of the most serious complication of preeclampsia (PE), i.e., a prothrombotic state that can lead to ischemic heart disease, stroke, and venous thromboembolism, especially in those with pre-existing medical conditions [9]. Studying the impact of oxidative stress on hemostatic actions will help identify key targets for prediction, prophylaxis, and treatment of PE.

Cancer initiation and progression have been associated with oxidative-stress-enhanced DNA damage. In turn, the high metabolism of cancer cells is associated with an increase in ROS production. Consequently, cancer cells develop resistance to oxidative stress as one of their major adaptive changes. Peroni et al. reported the resistance of the human astrocytoma ADF cells to oxidative stress, with specific regard to the enzymatic activities involved in 4-hydroxynonenal (HNE) removal. ADF cells counteracted oxidative stress conditions better than normal cells, thus confirming the redox adaptation demonstrated for several cancer cells [10]. The clarification of this aspect may indicate new enzymatic targets to be inhibited to antagonize astrocytoma cells survival.

Gaikwad and Srivastava highlighted that ROS play a dual role in cancer; below certain threshold values, ROS act as signaling molecules leading to activation of oncogenic pathways. However, high levels of ROS exhibit an anti-tumoral effect leading to cellular death through various programmed cell death pathways including apoptosis, autophagy, ferroptosis, pyroptosis, and anoikis. Phytochemicals can stimulate ROS accumulation beyond the threshold value and could, therefore, be considered as part of a therapeutic strategy to promote programmed cell death in cancer [11].

Antineoplastic agents induce oxidative stress in cancer as well as in normal cells. Accordingly, ROS production and consequent impairment of mitochondrial function substantially contribute to cisplatin nephrotoxicity. The scaffolding protein Na^+^/H^+^ exchanger regulatory factor 1 (NHERF1) plays a fundamental role in the maintenance of a proper kidney ion transport and metabolism. Bushau-Sprinkle and co-authors showed that NHERF1 loss imposes a shift in cell metabolism towards the pentose phosphate pathway, which may sensitize kidney cells to the oxidative stress caused by cisplatin. These findings identify novel potential biomarkers and therapeutic targets in the detection and prevention of cisplatin-induced nephrotoxicity [12].

Oxidative stress plays a fundamental role in aging. In this regard, age-related macular degeneration (AMD) has been considered by Ulanczyk et al. Significant alterations in the levels of several crucial antioxidant enzymes in red blood cells and platelets from patients with AMD have been observed, indicating that the equilibrium of the endogenous antioxidant system is disrupted in AMD. A diet rich in green vegetables, fish, and omega-3-rich oils, together with physical exercise, might delay disease progression and help retain a better visual function in patients with AMD [13].

Elevated oxidative stress represents a striking aggravating factor of retinal dystrophies, including age-related macular degeneration (AMD) and retinitis pigmentosa (RP). Donato and co-authors analyzed genes differentially expressed and differentially alternatively spliced in human retinal pigment epithelium cells exposed to the oxidant agent N-retinylidene-N-retinylethanolamine (A2E) or left untreated. This study identifies novel pathways involved in the oxidative-stress-induced etiopathogenesis of retinal dystrophies [14].

The elderly are most likely the main population to be treated by stem cell therapy. Stem cells may be negatively influenced by oxidative stress in our body; therefore, the restoration of the cellular functions of stem cells injured by oxidative stress is crucial. In this context, rescue of adipose-derived stem cells (ADSCs) from diseased or aged patients and the modulation of their activity by substance P is anticipated to increase the therapeutic potential of stem cell transplantation [15].

Aging is also characterized by reduced immune response, a process known as immunosenescence. In particular, the immunological memory, which is a typical feature of the adaptive immune system, is impaired in the elderly. Oxidative stress was shown to negatively affect the maintenance of immunological memory in old age and promote the onset of a pro-inflammatory environment in the bone marrow [16]. Meryk et al. showed that the antioxidants vitamin C and N-acetylcysteine decreased oxidative stress as well as the expression of pro-inflammatory molecules in the bone marrow and spleen of aged mice, boosted the production of new memory T cells, and activated dendritic antigen-presenting cells. These findings suggest that the generation and maintenance of memory T cells in old age may be improved by targeting oxidative stress [17].

Continued oxidative stress can cause chronic inflammation. Cigarette smoke, which contains high concentrations of oxidants, promotes chronic inflammation in the airways, and is involved in the pathogenesis of chronic rhinosinusitis (CRS). In CRS, excessive production of ROS not only affects the inflammatory response but also leads to tissue remodeling. Park et al. demonstrated the role of the ROS/PI3K/Akt and NF-κB signaling pathways in mediating cigarette smoke extract-stimulated MMP-2/TIMP-2 imbalance in nasal fibroblasts, which might contribute to tissue remodeling in CRS [18].

Lazado et al. focused on the use of oxidative chemical compounds in several husbandry practices, to understand how chemical stressors impact fish physiology, especially at mucosal surfaces. Their study revealed the interplay between oxidative stress and the nasal microenvironment of Atlantic salmon at the molecular and cellular level. The results offer insights into the oxidative stress responses at the nasal olfactory mucosa, which is considered the most ancient arm of the mucosal immune system in vertebrates [19]. From a practical perspective, data suggest that some of the oxidant chemicals commonly used in aquaculture could trigger oxidative stress that, if chronic, may be detrimental to fish health.

“An Update of Palmitoylethanolamide and Luteolin Effects in Preclinical and Clinical Studies of Neuroinflammatory Events” focuses on neuroinflammation. The review analyzes the key role of N-palmitoylethanolamine that, though lacking in antioxidant effects, exhibits powerful neuroprotective and anti-inflammatory properties and its co-ultramicronization with the flavonoid luteolin, which makes it more effective than the molecule alone [20].

Vascular permeability is increased in inflammation. Freitas et al. studied the impact of free radical generation on microvascular permeability. These authors showed that bradykinin and histamine utilize different signaling pathways to increase microvascular permeability, i.e., ROS and nitric oxide, respectively, and cytokines may potentiate the bradykinin effect. Importantly, animals treated with simvastatin did not display potentiation of bradykinin-induced microvascular permeability by cytokines. This finding underpins the anti-inflammatory properties of statins, which may be directly associated with their cellular antioxidant activities [21].

Acute pancreatitis is an inflammatory process of the pancreatic tissue that may lead to liver injury, and obesity is a risk factor for the development of hepatic complications in the context of acute pancreatitis. Rius-Pérez and collaborators showed that pancreatitis leads to marked induction of the transcriptional co-activator PGC-1α in the mouse liver, with consequent protection from nitrosative stress. Obesity caused PGC-1α deficiency and enhanced nitrosative stress in the liver during pancreatitis. These findings underscore a novel protective role of PGC-1α in preventing nitrosative stress in the liver during the development of acute pancreatitis [22].

Epigenetic mechanisms regulate the expression of genes involved in inflammation. Phenolic compounds, which are very well known for their antioxidant properties, have the ability to modulate gene expression through the regulation of epigenetic mechanisms, including DNA methylation, histone modification, and miRNA expression. Ciz and co-authors reviewed these aspects and suggested that targeting epigenetics by dietary polyphenolic compounds may represent an attractive strategy in the prevention and treatment of inflammatory conditions [23].

The use of natural antioxidants and nutraceuticals in the context of oxidative-stress-related diseases are the subject of intense investigation. Among natural antioxidants with beneficial effects against inflammation, cashew (*Anacardium occidentale* L.) nuts improve the endogenous antioxidant activity and limit pro-inflammatory cytokines release [24]. This study increases the knowledge on the role of foods in the modulation of oxidative stress and inflammation in an in vivo experimental model, thus opening the way to new investigations on the benefits of a balanced diet in ensuring optimal health.

Rizzo reviewed the published clinical trials on the possible antioxidant effect of soy, soy foods, and soy bioactive substances. This author denoted that, despite the generally accepted beneficial role of soy on human health, its antioxidant properties have not been unequivocally demonstrated and deserve to be further investigated [25].

A diet enriched in legumes is fundamental to positively modulate redox balance, lipid metabolism, and insulin sensitivity. Centrone and co-authors studied the nutraceutical features of two genetically and phenotypically distinct chickpea cultivars, i.e., MG_13 and PI358934. Both chickpea accessions showed significant antioxidant ability; however, only MG_13 reduced the lipid over-accumulation in steatotic rat hepatoma cells and in the liver of mice fed on a high-fat diet. These findings underscore the importance of studies characterizing the distinct effects and precise composition of nutraceuticals [26].

Although widely recognized as beneficial for human health, antioxidants may exhibit toxic pro-oxidant effects following reaction with reactive chemical species. Giordano and co-authors showed that Trolox (6-hydroxy-2,5,7,8-tetramethylchroman-2-carboxylic acid), a hydrophilic analog of vitamin E well known for its strong antioxidant activity, develops pro-oxidant properties in HeLa cells when used at concentrations higher than 20 µM and leads to apoptotic cell death. This study denotes that the antioxidant activity of bioactive compounds may have a concentration threshold that must be carefully considered in the context of an antioxidant therapy [27].
